# Esports Fan Engagement: A Comparison of PC and Console Esports Team Fans

**DOI:** 10.3389/fspor.2022.880294

**Published:** 2022-04-29

**Authors:** Marcel Huettermann, Anthony D. Pizzo

**Affiliations:** ^1^School of Management and Law, Institute of Marketing Management, ZHAW Zurich University of Applied Sciences, Winterthur, Switzerland; ^2^School of Business, Management and Leadership Department, La Salle University School of Business, Philadelphia, PA, United States

**Keywords:** esports, fans, engagement, consumer behavior, sports, digitalization, technology, marketing

## Abstract

Esports, competitive video game competitions, are a leading digital innovation at the nexus of sports, business, and technology. Given their prominent position, esports have received extensive media and academic attention. In particular, esports fans, primarily tech-savvy and affluent young adults, have been the foci of this attention. Accordingly, a large number of studies has centered on these influential consumers, examining their motives to spectate, support, and follow esports teams and players. To date, esports have been examined very broadly, neglecting differences in the multitude of games, genres, and platforms which influence their consumption. In particular, the platform (or medium), plays a substantial role in how consumers engage with esports teams and players. These platforms include personal computers (PCs) and video gaming consoles. The purpose of this study is to identify differences in how fans of PC and console based esports teams engage with their favorite esports team. We collected data from both PC and console esports team fans *via* an online survey (*N* = 514), analyzing said data using structural equation modeling and multigroup analysis. Our results highlight that fans of console-based esports teams value both emotional engagement and management cooperation, underscoring the more intimate and personal experience afforded by consoles (vis-à-vis PCs). Overall, our study elucidates differences in esports fan engagement and helps to further identify critical differences that influence esports consumer behavior.

## Introduction

Digital innovations help to create opportunities for firms to engage with new markets (Rachinger et al., 2019). Digital innovations include novel and disruptive technologies such as artificial intelligence, blockchain, Internet-of-things, the Metaverse, and non-fungible tokens (Lopez et al., 2021). A leading disruptive innovation at the center of sports, business, and technology is esports (Scholz, 2019).

Esports are competitive video game competitions (Pizzo et al., 2018). They have their origins in South Korean PC bangs and grown in popularity, with hundreds of millions of global fans, spectators, and participants (Funk et al., 2018). In particular, the appeal of esports stems from their consumers. Esports consumers are primarily affluent and hard-to-reach young adults, a coveted target market for many traditional businesses and organizations (Huettermann et al., 2020). Consequently, esports have attracted substantial media visibility and financial investment, particularly among professional and amateur sport leagues and teams seeking to connect with, and capitalize on, this lucrative market segment (Pizzo et al., 2019).

Given their position at the nexus of multiple domains, esports have attracted considerable academic attention (Cranmer et al., 2021). To better understand esports, scholars have drawn parallels between sport and esports consumers as a way of making sense (or sensemaking) of them and their novelty (Pizzo et al., 2021). This line of scholarship finds that consumers of each share many similar consumption motives to watch (spectate) and play (participate) in competitive video gaming (e.g., Pizzo et al., 2018; Jang and Byon, 2020, 2021; Qian et al., 2020; Tang et al., 2020). While the role and status of esports as a form of sport (or not) will likely always be debated (Funk et al., 2018; Scholz et al., 2021), there are substantial economic (Scelles et al., 2021), managerial (e.g., team and player management), operational (e.g., event hosting), and marketing (e.g., team rivalries) similarities between them (Funk et al., 2018).

The study of esports has begun to evolve beyond broad conceptualizations of esports and their consumers. Scholars are beginning to recognize that—despite fragmented governance (Peng et al., 2020)—esports have become increasingly popular, structured, and organized, and broad conceptualizations of esports are problematic (e.g., Ji and Hanna, 2020; Rogers et al., 2020). For instance, there is a “wide variety of esports titles, with little known about the similarities and differences…of their audiences” (Baker and Pizzo, 2021, p. 2). Indeed, esports encompass a wide variety of genres, such as battle royale, digital card collectible games, multiplayer online battle arenas (MOBAs), first-person shooters (FPS), real-time strategy (RTS), sport simulations (e.g., FIFA, NFL, and NBA annual titles), among various other genres. Moreover, advances in digital innovations and technologies have provided a variety of platforms (or mediums) to consume esports related content.

Esports are watched and played on personal computers (PCs), video game consoles (e.g., Nintendo Switch, PlayStation, and Xbox consoles), and mobile devices. These platforms provide unique and diverse ways to engage with competitive video gaming content, helping to further increase the popularity of esports. Moreover, esports team-based competitions primarily use PCs and video gaming consoles (cf. mobile devices), as these platforms are better suited to team competition, whereas mobile devices generally involve two individuals competing against one another (Esports Insider, 2021). Yet despite the popularity of PC and console based esports, most esports consumer behavior studies have focused on PC based gaming (Jang and Byon, 2020), neglecting the growing console-based esports market.

The purpose of this study is to identify differences in how fans of PC and console based esports teams engage with esports teams. By addressing our study purpose, we advance esports scholarship and break down their broad conceptualization by unpacking salient differences in the fan engagement factors that are distinct to PC and console based esports team fans. The following content reviews relevant literature related to fans, fan engagement, PC and video game consoles, and is followed by our methods, results, discussion, and broader implications of our study.

### Literature Review

Fans are an essential part of sport and esports. A fan is a consumer of a good or service who has an emotional connection with a sport entity (Hunt et al., 1999), such as a league, team, player, or other fans. Fans tend to have long-term and highly committed relationships with the teams they follow (Funk et al., 2016). Fans differ from spectators as being a fan involves a higher level of excitement, emotion, and intensity (Hirt et al., 1992). In short, fans are essential to a team's long-term success and sustainability as they are more engaged than causal spectators.

Fan engagement is critical to teams and players. Fan engagement is defined as “a concept to reflects fan's involvement with a sports team or with other fans of the sport team” (Huettermann and Kunkel, 2022, p. 3). This involvement includes both non-transactional and transactional behavior. In our study, given the digital nature of esports and the strong online communities surrounding them, we focus on non-transactional behaviors. Within esports, many of the transactional involvement components and related opportunities (e.g., merchandising, event ticketing) are less prevalent than in professional sports, underscoring the importance of non-transactional involvement (Mangeloja, 2019). Esports teams are generally not connected with a physical location. This is in contrast to traditional sports where teams have a physical connection to a city or geographic region. For instance, the National Basketball Association (NBA) is a professional North American sports league with 30 teams located within major metropolitan areas, such as the Los Angeles Lakers, Chicago Bulls, Boston Celtics, and New York Knicks (Lopez et al., 2021). This is in contrast to many esports teams which lack an inherent geographic tie in, such as leading esports teams (in terms of overall team earnings) of FaZe Clan, Team Liquid, Evil Geniuses, 100 Thieves, and Gen.G (Settimi, 2020).

To better understand the non-transactional engagement factors germane to esports consumers, we assessed the non-transactional engagement factors identified by Huettermann and Kunkel (2022). These factors include: Management cooperation, emotional engagement, word-of-mouth, knowledge generation, and socialization, with their relationship to behavioral intentions. Non-transactional behaviors play a salient role on behavioral intentions. Management cooperation reflects the attitude of fans who actively contribute to the administrative management of a team to ensure its success (Yoshida et al., 2014). For instance, fans can give feedback to team management and actively participate in the design of products and services in a value cocreation process (Kumar et al., 2010). Emotional engagement is the affective commitment of a fan and is based on feelings of identification, loyalty, and affiliation (Verhoef et al., 2002). Higher levels of emotional engagement result in a multitude of increased loyalty and consumption behaviors, such as increased merchandise purchases and event patronage (Funk et al., 2016). Word-of-mouth (WOM) involves the non-commercial communications about a company's products or services (Arndt, 1967). WOM marketing is an influential form of sport marketing, as sport fans actively engage in discussions about the management and performance of sport teams (Uhrich, 2014; Kunkel et al., 2017). Knowledge generation is the knowledge created and acquired by fans based on their interactions with a sport entity (Hibbert et al., 2012; Brodie et al., 2015; Hollebeek et al., 2019), particularly in relation to team sports (Huettermann et al., 2019). Socialization involves the informal interactions among sport fans. Notably, sport fans frequently discuss sport events before, during and after games, about a wide variety of topics, such as past results, performances, and management decisions (Huettermann and Kunkel, 2022). The social aspect of sports provides fans with a sense of belonging and identification (Crawford, 2004; Funk et al., 2016).

Fan engagement is a critical component of esports. Esports team owners and managers recognize that engaging their fans requires additional strategic considerations to connect with a globally dispersed audience (Pizzo et al., 2021). Notably, several prominent esports leagues, namely the League of Legends Champion Series (LCS) and Overwatch League (OWL), have mimicked traditional sport franchise models. These models embrace the geographic ties in used in traditional sport. For instance, OWL franchises include teams such as the Seoul Dynasty, Shanghai Dragons, Vancouver Titans, and Philadelphia Fusion (Pizzo et al., 2022a). Yet even for these geo-based (or regional) esports leagues and teams, team owners acknowledge that building an organic connection with local fans is difficult, as both LCS and OWL competitions are primarily held in centralized locations outside the home market for these teams (Bailey, 2018). This diminishes opportunities to capitalize on transactional opportunities, further underscoring the importance of identifying relevant fan engagement factors (Huettermann and Kunkel, 2022).

Furthermore, research on esports fan engagement is limited. While insightful, existing esports scholarship treats esports as a monolithic concept (Scholz, 2019). This omits opportunities to unpack the nuances that permeate the various video game publishers, genres, platforms, online communities, and other factors which influence esports fan engagement (Baker and Pizzo, 2021). Given the importance of teams for PC and video game console based esports, we focus on fans of esports teams which compete on these platforms. While the majority of esports competitions are held using PCs, consoles are a fast-growing segment of the esports industry, with the growth fueled by next generation consoles such as the PlayStation 5 and Xbox Series X.

Relative to PC gaming, video game consoles provide distinctive gaming experiences. PC gaming focuses more on the latest and most powerful high-end computers which generally have superior application and graphic processing power (Pickell, 2019). By contrast, video game consoles provide a lower price point and increased ease-of-use, but with less flexibility and game options than their PC counterparts (Pickell, 2019). In general, PC gaming offers a wider selection of games and superior graphics, with console gaming easier to use, offering a more casual gaming experience (Spohn, 2021).

From a consumer behavior perspective, individuals exhibit clear preferences in the esports teams they follow based on their preferred video gaming platform. Dedicated online communities exist on Twitch and Discord (leading video gaming streaming and communication platforms) for PC and console team fans. Moreover, esports video game console-based teams, players, and streamers are becoming increasingly popular, leveraging functions built directly into consoles that allow them to stream directly from their console (Middler, 2021). Furthermore, cloud-based services, such as Nvidia's GeForce Now, allow gamers to stream and play games across a variety of devices, from PCs to mobile devices, further expanding the popularity of competitive video gaming beyond PCs (Henderson, 2021).

Overall, esports provide a novel way to engage audiences. With increased connectivity across video gaming platforms, console-based esports and related teams are becoming more popular. Yet fan engagement is still a major concern in esports, with extant academic research primarily focusing on PC based esports and related consumption. The exploratory nature of our study seeks to address the limitations of existing esports research and elucidate upon key differences in esports consumer behavior (i.e., PC vs. console-based esports fans) by answering the following research question:

How do fan engagement factors differ between PC and console-based esports teams?

## Materials and Methodology

### Measures

To answer our research question, we adapted the fan engagement model of Huettermann and Kunkel (2022). The model integrates established fan engagement constructs of: Management cooperation (MC), emotional engagement (EE), knowledge generation (KG), socialization (S), word-of-mouth (WOM), and intentions (I). The model integrates the constructs of management cooperation, emotional engagement, and word-of-mouth from Yoshida et al. (2014). The constructs of knowledge generation and socialization were integrated and adapted from Trail and James (2001). The intention construct was adapted from Hedlund (2019). The model integrates established fan engagement constructs into a parsimonious tool which can examine the relationship between fan engagement and behavioral intentions (Huettermann and Kunkel, 2022). Overall, the model can inform esports team marketing fan engagement practices. All of the items in the model (excluding demographics) were measured on a 5-point Likert scale, ranging from 1 = *strongly disagree* to 5 = *strongly agree*. Table 1 provides an overview and visualization of our study's measures and relationships.

**Table 1:** Constructs and items.

**Construct/item**	**References**
**Intentions**	Hedlund, 2019
*I will attend my esports team's games in the future*.	
*I will buy merchandise of my esports team in the future*.	
*I will read stories in the media about my esports team in the future*.	
**Management cooperation**	Yoshida et al., 2014
*I try to work cooperatively with my esports team*.	
*I do things to make my esports team's event management easier*.	
*The employees of my esports team get my full cooperation*.
**Emotional engagement**	Yoshida et al., 2014
*Watching games of my esports team makes me happy*.	
*Watching games of my esports team gives me pleasure*.	
*I feel good when I watch games of my esports team*.
**Knowledge generation**	Trail and James, 2001
*I regularly track the statistics of specific esports players*.	
*I usually know my esports team's win/loss record*.	
*I read my esports team's scores and statistics regularly*.	
**Socialization**	Trail and James, 2001
*Interacting with other fans online is a very important part of watching games of my esports team*.	
*I like to talk to other people during the games of my team*.	
*Games are great opportunities to socialize with other people*.
**Word of mouth**	Yoshida et al., 2014
*I often interact with other fans to talk about issues related to my esports team*.	
*I often advise other fans on how to support my esports team*.	
*I spend time on social media sharing information with other fans of my esports team*.	

### Data Collection

We collected data from European esports fans (*N* = 514) who indicated they were fans of either a PC or console-based esports team. Online questionnaires were developed in English and sent to esports fans *via* direct messages to followers of Twitch channels for various leading esports teams. To encourage individuals to take part in the survey, participants were given the opportunity to win one of five a gift cards for a large, online retailer. We ensured that each participant could complete the survey only once. Of the participants, 66.3% (*n* = 341) were men and 33.7% (*n* = 173) were women. Most were between the ages of 30–44 (47.5%, *n* = 244) or under 30 (43.2%, *n* = 222), with the rest between the ages of 45–59 (7.4%, *n* = 38) or 60 and over (1.9%, *n* = 10). Most participants had a full-time job (54.7%, *n* = 281). Finally, 53.7% (*n* = 276) followed a PC esports team, while 46.3% (*n* = 238) followed an esports console-based team. Table 2 provides an overview of the demographic characteristics of our sample, including breakdowns for PC and console team fans.

**Table 2:** Demographic characteristics.

**Characteristic**	**Details**	**Total**	**Percentage**	**PC gamers** **(*n*)**	**PC gamers** **(%)**	**Console gamers** **(*n*)**	**Console gamers** **(%)**
Gender	Male	341	66.3%	189	68.5%	152	63.9%
	Female	173	33.7%	87	31.5%	86	36.1%
	*Total*	*514*	*100%*	*276*	*100%*	*238*	*100%*
Age	16–29	222	43.2%	119	43.1%	103	43.3%
	30–44	244	47.5%	118	42.8%	126	52.9%
	45–59	38	7.4%	29	10.5%	9	3.8%
	≥60	10	1.9%	10	3.6%	0	0.0%
	*Total*	*514*	*100%*	*276*	*100%*	*238*	*100%*
Employment status	Full-time job	281	54.7%	146	52.9%	135	56.7%
	Part-time job	67	13.0%	35	12.7%	32	13.5%
	Student	67	13.0%	36	13.0%	31	13.0%
	Not currently employed	20	3.9%	12	4.4%	8	3.4%
	Other	79	15.4%	47	17.0%	32	13.5%
	*Total*	*514*	*100%*	*276*	*100%*	*238*	*100%*
Platform	PC	276	53.7%	276	100.0%	0	0.0%
	Console	238	46.3%	0	0.00%	238	100.0%
	*Total*	*514*	*100%*	*276*	*100%*	*238*	*100%*

### Data Analysis

We used IBM SPSS Statistics 28 and IBM SPSS AMOS 28 for data analysis. A data cleaning procedure was applied in which the following cases were removed: (1) incomplete questionnaires, (2) questionnaires completed in an unrealistically short time, (3) questionnaires in which the same answer had been checked for each question or *straightlining* (Rossi et al., 2013). After this, a total of 514 surveys (out of 556) were used for analysis. Following Brown's approach (2006) we tested the reliability and validity of the measures using confirmatory factor analysis (CFA). After this, we used structural equation modeling (SEM) to test the proposed model. A multigroup analysis was conducted to investigate differences in fan engagement based on the esports team platform (either PC or console). For the evaluation of the overall model, we used established criteria according to Byrne (2006) and Hair et al. (2014).

## Results

### Reliability and Validity Testing

Results from the CFA indicated that the measures met the criteria proposed by Hair et al. (2014) and Hu and Bentler (1999) and provided a good model fit (RMSEA = 0.072; χ^2^/df = 2.861; *p* < 0.01; SRMR = 0.0405, NFI = 0.928; CFI = 0.951; TLI = 0.938; and IFI = 0.925).^1^ All reliability indicators (IR) were above 0.40 (Bagozzi and Baumgartner, 1994) and all factor reliability indicators were above 0.60 (Bagozzi and Yi, 1988). Linearity was assessed through the examination of correlation coefficients among constructs. Specifically, absolute values of correlation coefficients of <0.85 are adequate for statistical analysis (Kline, 2011). Normality, multicollinearity, and outliers were tested based on the criteria established by Hair et al. (2014). The results of our reliability testing ensured that data met the assumptions for structural equation modeling (Kline, 2011; Hair et al., 2014). The mean scores, factor loadings, standard deviation, and the average variance explained (AVE) values for the six-factor solution are presented in Table 3.

**Table 3:** Confirmatory factory analysis (CFA) results.

**Factor/item**	**Mean score**	**SD**	**Standardized factor loading**	**AVE**
**Intentions**				0.556
*I will attend my esports team's games in the future*.	2.25	1.230	0.755	
*I will buy merchandise of my esports team in the future*.	2.79	1.258	0.769	
*I will read stories in the media about my esports team in the future*.	2.92	1.266	0.712	
**Management cooperation**				0.665
*I try to work cooperatively with my esports team*.	2.26	1.200	0.853	
*I do things to make my esports team's event management easier*.	2.07	1.251	0.786	
*The employees of my esports team get my full cooperation*.	2.67	1.271	0.806	
**Emotional engagement**				0.769
*Watching games of my esports team makes me happy*.	2.84	1.247	0.901	
*Watching games of my esports team gives me pleasure*.	3.01	1.252	0.858	
*I feel good when I watch games of my esports team*.	2.86	1.238	0.872	
**Knowledge generation**				0.772
*I regularly track the statistics of specific esports players*.	2.54	1.279	0.846	
*I usually know my esports team's win/loss record*.	2.42	1.253	0.869	
*I read my esports team's scores and statistics regularly*.	2.47	1.263	0.919	
**Socialization**				0.631
*Interacting with other fans online is a very important part of watching games of my esports team*.	2.67	1.246	0.751	
*I like to talk to other people during the games of my team*.	2.61	1.260	0.842	
*Games are great opportunities to socialize with other people*.	2.75	1.258	0.788	
**Word of mouth**				0.733
*I often interact with other fans to talk about issues related to my esports team*.	2.73	1.238	0.805	
*I often advise other fans on how to support my esports team*.	2.80	1.271	0.870	
*I spend time on social media sharing information with other fans of my esports team*.	2.67	1.281	0.891	

*AVE, Average variance explained; SD, standard deviation*.

AVE values for all constructs met the recommended threshold of 0.50 (Fornell and Larker, 1981), and all Cronbach's alpha values were above 0.70, confirming the internal consistency of the six constructs (Nunnally and Bernstein, 1994). Discriminant validity between the six dimensions was confirmed by AVE values that were above the squared correlations between the constructs. The correlation matrix for the six constructs is shown in Table 4.

**Table 4:** Correlation matrix (CFA).

	**AVE**	**I**	**MC**	**EE**	**KG**	**S**	**WOM**
Intentions (I)	0.56	**1.00**	0.37	0.46	0.37	0.28	0.44
Management cooperation (MC)	0.67	0.61	**1.00**	0.26	0.21	0.11	0.25
Emotional engagement (EE)	0.77	0.68	0.51	**1.00**	0.12	0.16	0.22
Knowledge generation (KG)	0.77	0.61	0.46	0.35	**1.00**	0.10	0.16
Socialization (S)	0.59	0.53	0.34	0.40	0.31	**1.00**	0.23
Word of mouth (WOM)	0.73	0.66	0.50	0.47	0.40	0.48	**1.00**

*Below the values in bold are correlation estimates. Above the values in bold are squared correlation estimates*.

### Structural Equation Model Testing

**Figure 1:**
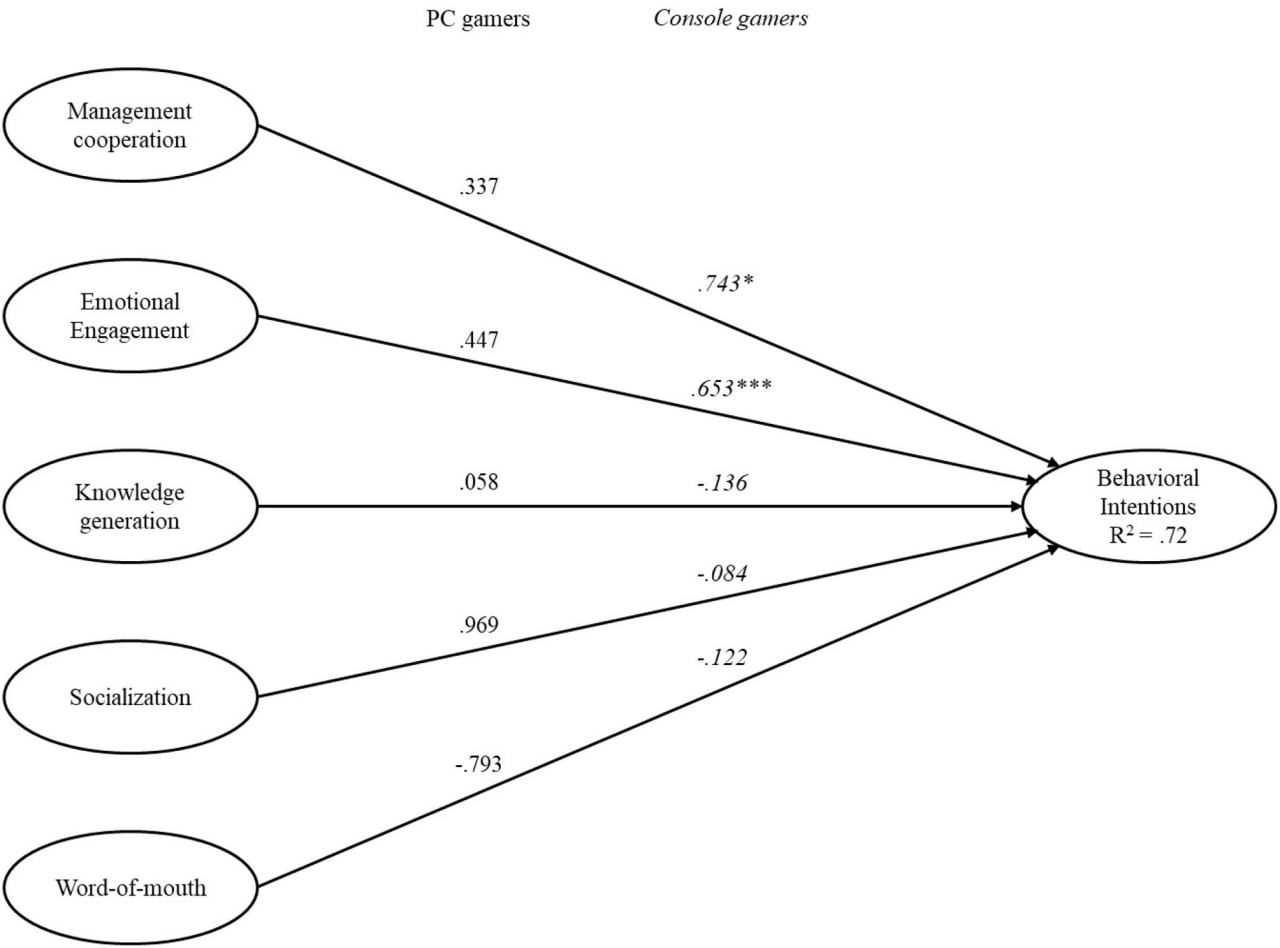
Relationship between fan engagement and behavioral intentions. *Note*. Values in italics are for console gamers. Values not italicized are for PC gamers. ***p < 0.001, *p < 0.05.

The SEM results indicated that the conceptual model showed a good fit (RMSEA = 0.076; χ^2^/df = 2.972; *p* < 0.01; SRMR = 0.0349, NFI = 0.946; CFI = 0.958; TLI = 0.947; and IFI = 0.931). Results indicate a significant positive relationship for emotional engagement (β = 0.639, *p* < 0.001) and a near significant relationship for management cooperation (β = 0.591, *p* = 0.059). Knowledge generation (β = 0.016, *p* = 0.951), word of mouth (β = −0.140, *p* = 0.818), and socialization (β = −0.059, *p* = 0.884) were insignificant. Results are shown in Table 5 and visualized in Figure 1. All reported results were considered significant at the 5% level.

**Table 5:** Structural equation model (SEM) results.

**Path**			**Standardized coefficient (β)**	**SE**	***t***	***p***
Management cooperation	→	Intentions	0.591	0.300	1.887	0.059
Emotional Engagement	→		0.639	0.146	3.795	<0.001
Knowledge generation	→		0.016	0.135	0.107	0.915
Socialization	→		−0.059	0.399	−0.146	0.884
Word of mouth	→		−0.140	0.527	−0.230	0.818

*SE, standard error*.

### Multigroup Analysis

To test the applicability of the proposed model across groups, a multigroup analysis (MGA) was conducted for model stability (Byrne et al., 1989). MGA provides a separate analysis of path structure for each group (i.e., PC and video game console gamers) since the estimation of the base model does not impose constraints between groups (Byrne, 2004). Therefore, MGA was deemed an appropriate method to address our research question. The sample was divided into two groups: those who follow either a PC (*n* = 276) or video game console based esports team (*n* = 238) to understand the differences between the gaming platforms. The MGA results indicated that the conceptual model showed good fit (RMSEA = 0.059; χ^2^/df = 2.784; *p* < 0.01; SRMR = 0.0405, NFI = 0.923; CFI = 0.949; TLI = 0.935; and IFI = 0.949). However, in contrast to the single-group analysis, the results of the MGA showed distinct results. We found that emotional engagement (β = 0.653, *p* < 0.001) and management cooperation (β = 0.743, *p* = 0.026) were significant for video game console team fans, but not for PC team fans (emotional engagement: β = 0.447, *p* = 0.209; management cooperation: β = 0.337, *p* = 0.599). Other variables were not significant. Results of the MGA are shown in Table 6. All reported results were considered significant at the 5% level.

**Table 6:** Multigroup analysis (MGA) results.

**Path**			**Standardized coefficient (β)**		**SE**		***t***		***p***	
			**Console**	**PC**	**Console**	**PC**	**Console**	**PC**	**Console**	**PC**
Management cooperation	→	Intentions	0.743	0.337	0.298	0.636	2.224	0.526	0.026	0.599
Emotional Engagement	→		0.653	0.447	0.146	0.313	3.801	1.256	<0.001	0.209
Knowledge generation	→		−0.136	0.058	0.171	0.404	−0.671	0.128	0.502	0.898
Socialization	→		−0.084	0.969	0.392	0.794	−0.220	1.316	0.826	0.188
Word of mouth	→		−0.122	−0.793	0.469	0.825	−0.220	−0.842	0.826	0.400

*SE, standard error*.

## Discussion

### Key Results

Overall, our findings underscore the importance of emotional engagement and management cooperation for esports video game console team fans. Console-based fans exhibit higher levels of emotional engagement relative to their PC-based counterparts. There are a variety of ways to explain this finding, but we posit that this stems from the intrinsic pleasure and personalized experience provided by consoles. The culture surrounding esports and competitive video gameplay has been described as toxic, particularly in online play (Kordyaka et al., 2020), a hallmark of PC-gaming (Ruvalcaba et al., 2018). Video game consoles provide a viable alternative to PCs gaming and are also less reliant on in-game communications than their PC counterparts. While many esports which rely on in-game communication are cross platform (e.g., Fortnite), many individuals are metaphorically pushed into “inferior” gaming platforms, such as mobile devices (Paaßen et al., 2017), as the advanced equipment and hardware used by PC gamers has created an extremely competitive culture. By contrast, console games offer more leisurely experiences, and as a byproduct, likely foster a more intimate and personally engaging experience for console based esports teams and their fans. For instance, video game consoles are easy to use, do not require hardware upgrades, and facilitate multiplayer with friends who own consoles (Crucial, 2021), appealing to the growing number of casual female competitive gamers (Jang and Byon, 2021).

Management cooperation was also significantly and positively associated with the behavioral intentions of esports video game console team fans. This finding is intriguing as it indicates that video game console based esports team fans value actively contributing to the management and value cocreation of their team. By comparison, PC-based fans did not. Console team fans desire to contribute to the management and success of their team more actively, underscoring how console based esports teams have a more engaged audience. Thus, the popularity of PC based esports (and teams) may be a double ended sword. From one perspective, these teams benefit from large and global fan bases (Funk et al., 2018), yet their popularity may provide less intimate and thereby less engaging experiences offered by their console-based counterparts. Indeed, while there is intense competition and cross-platform play across PCs and consoles, PC games tend to have a have a longer shelf life than console games, as popular console games such as FIFA or Call of Duty have new editions each year (Copenhaver and Griffin, 2021). Whereas, PC games are often maintained—independent of video developers and producers—by game modders. Video game modding (short for modification) refers to altering how a game looks or behaviors, with the intent to extend the replay and hedonic value of a game (Poor, 2014). As such, video game console based esports team fans are more likely to want to support their favorite game developers and producer, whereas PC based gamers are more independent from developers/producers.

Notably, we found that for both PC and video game console esports team fans, knowledge generation, WOM, and socialization were not significantly related to behavioral intentions. This finding underscores that engaging esports consumers—independent of platform—remains an area in need of further inquiry. Indeed, scholars are beginning to call the identification and development of factors distinct to esports (cf. traditional sports) to better understand esports consumers behavior (Qian et al., 2020). Specifically, our non-significant findings suggest that established scales and constructs from sport management may not be particularly insightful to understand esports consumers behavior. Similar sentiments have been expressed by other scholars calling for a more distinctive analysis and understanding of esports (Wood et al., 2019) and its consumers (e.g., Qian et al., 2020).

### Limitations and Future Research

Our research is subject to four primary limitations. First, we focused on PC and console gaming. This overlooks the rapidly growing importance of mobile gaming. Mobile gaming offers unparalleled levels of access, such as gaming on the go, as well as the benefits of technology leapfrogging (Seo et al., 2019). Many developing countries do not have the required resources and infrastructure to support PC and console-based competitions, yet mobile devices are helping to bridge the digital divide. As such, mobile gaming promises to be the next frontier in esports and related competitions, yet there is a distinct lack of scholarly inquires into the benefits of the medium.

Second, we focused on differences in the gaming platform, overlooking factors such as game genre and publishers, among other factors which influence esports related consumption (Baker and Pizzo, 2021). Furthermore, we did not distinguish between different types of video game consoles. There are a variety of consoles, including PlayStation, Xbox, and Nintendo Switch, as well as cloud-based applications such as Amazon Luna and Google Stadia which stream games directly to players. There are likely additional engagement strategies salient to these streaming services, as they generally have a fundamentally different business model, namely gaming-as-a-service (GaaS). Accordingly, this limitation provides opportunities for future inquires to further theorize and examine if and how differences in platforms, game genre, etc. influence related consumption behaviors.

Third, we focused on esports, neglecting the broader importance of the video games industry. Despite the rapid growth of esports, it is pertinent to note that esports encompass only a small portion of the larger video game industry. While estimates vary, the video game industry is projected to generate between $150 and $200 billion in revenue in 2023 (Accenture, 2021). By contrast, the esports marketspace is expected to generate $1.3 to $1.5 billion in 2023 (Accenture, 2021). To put these figures in perspective, by the most liberal estimates, esports constitute at most 1% of the total video game market. Yet esports are disproportionally the topic of media and academic inquiries alike, with commentators and scholars often erroneously using the terms *esports* and *video games* (or *video gaming*) interchangeably. The various financial estimates and interrelated nature of esports and video gaming suggests that the esports industry may likely be undervalued (Ahn et al., 2020). Accordingly, we suggest that future scholarly inquires further delineate between esports and video gaming to better understand the distinctive characteristics of these markets and related consumption practices. For instance, many individuals do not enjoy the competitive aspects of video gaming, instead they prefer non-competitive video gaming. Despite their aversion to the competitive elements of video gaming, these individuals still present distinct marketing opportunities, and their study can also enhance esports related research by identifying strategies to make competitive gaming more welcoming. Indeed, this sentiment is echoed in our findings, as video game console based esports (and related teams) offer a more personalized and intimate gaming experience than PC gaming.

Fourth, our conceptual model (Huettermann and Kunkel, 2022) focused on both attending events in-person and watching them online, neglecting the differentiating factors between physical attendance and online consumption. Distinguishing between these factors has become increasingly important, as some esports leagues, such as the OWL and LCS, have adopted regional franchise models from traditional sport, offering additional local event attendance and sponsorship activation opportunities (Jang and Byon, 2020). By contrast, other esports leagues, such as Valve's *The International*, based on the esport Dota 2 (Death of the Ancients), do not incorporate franchises into their business model. As such, future inquiries should account for the growing ways video game developers and producers structure their leagues to identify additional ways they can engage their audience, such as through dedicated esports venues (Jenny et al., 2018) which cater to the specific demands of esports consumers.

In addition, it is salient for future studies which adopt constructs and scales from other academic disciplines (e.g., sport management) to the study of esports to recognize that esports are a distinct activity. Esports incorporate elements from multiple areas (e.g., sport, business, leisure, information technology, management, hospitality, etc.) and their study will require scholars to understand not only their similarities with other disciplines, but their distinctive aspects as well (Pizzo et al., 2022b). This sentiment is echoed in our findings, as many sport fan engagement constructs were not directly applicable to esports team fans. Thus, the development of constructs and scales organic to esports (and their consumers) are increasingly to better understand this dynamic marketspace (Qian et al., 2020).

## Conclusion

Esports are a leading digitalization trend. They have attracted substantial academic and media attention and have become an integral part of mainstream society and culture. As the growth of esports continues, understanding the dynamics related to their consumption has become even more important (Baker and Pizzo, 2021; Jang and Byon, 2021). Accordingly, in our study, we identified differences in esports team fan engagement factors by platform, namely PC and video game console esports team fans. Our findings indicate that video game console esports team fans value emotional engagement and management cooperation, reflecting the intimate gaming experience offered by consoles, away from the limelight of PC-based teams, competitions, and their surrounding culture. Moving forward, scholars should continue to identify various other dynamics that influence esports related consumption, as well as further distinguish and delineate between esports and video gaming. In doing so, the growing academic and media attention given to esports can better target the needs of a rapidly growing industry and help sustain its growth and stability (Pizzo et al., 2022b).

## Data Availability Statement

The raw data supporting the conclusions of this article will be made available by the authors, without undue reservation.

## Ethics Statement

Ethical review and approval was not required for the study on human participants in accordance with the local legislation and institutional requirements. Written informed consent for participation was not required for this study in accordance with the national legislation and the institutional requirements.

## Author Contributions

All authors listed have made a substantial, direct, and intellectual contribution to the work and approved it for publication.

## Funding

Open access funding provided by ZHAW Zurich University of Applied Sciences.

## Conflict of Interest

The authors declare that the research was conducted in the absence of any commercial or financial relationships that could be construed as a potential conflict of interest.

## Publisher's Note

All claims expressed in this article are solely those of the authors and do not necessarily represent those of their affiliated organizations, or those of the publisher, the editors and the reviewers. Any product that may be evaluated in this article, or claim that may be made by its manufacturer, is not guaranteed or endorsed by the publisher.

## Footnotes

^1^RMSEA, Root Mean Square Error of Approximation; χ^2^/df, chi-square divided by the degrees of freedom; p, probability value; SRMR, Standardized Root Mean Square Residual; NFI, Normed Fit Index; CFI, Comparative Fit Index; TLI, Tucker-Lewis Index; and IFI, Incremental Fit Index.
